# Spatial distribution of rotavirus immunization coverage in Ethiopia: a geospatial analysis using the Bayesian approach

**DOI:** 10.1186/s12879-022-07825-1

**Published:** 2022-11-09

**Authors:** Kendalem Asmare Atalell, Alemneh Mekuriaw Liyew, Kefyalew Addis Alene

**Affiliations:** 1 Department of Pediatrics and Child Health Nursing, School of Nursing, College of Medicine and Health Sciences, University of Gondar, Gondar, Ethiopia; 2 Department of Epidemiology and Biostatistics, Institute of Public Health, College of Medicine and Health Sciences, University of Gondar, Gondar, Ethiopia; 3 Telethon Kids Institute, Nedlands, WA Australia; 4 Faculty of Health Sciences, Curtin University, Bentley, WA Australia

**Keywords:** Distributions, Ethiopia, Geospatial analysis, Immunization coverage, Rotavirus

## Abstract

**Introduction:**

Rotavirus causes substantial morbidity and mortality every year, particularly among under-five children. Despite Rotavirus immunization preventing severe diarrheal disease in children, the vaccination coverage remains inadequate in many African countries including Ethiopia. Measuring rotavirus immunization coverage in a lower geographic area can provide information for designing and implementing a targeted immunization campaign. This study aimed to investigate the spatial distributions of rotavirus immunization coverage in Ethiopia.

**Methods:**

Rotavirus immunization coverage data were obtained from the recent Ethiopian Demographic and Health Survey (EDHS 2019). Covariate data were assembled from different publicly available sources. A Bayesian geostatistics model was used to estimate the national rotavirus immunization coverage at a pixel level and to identify factors associated with the spatial clustering of immunization coverages.

**Result:**

The national rotavirus immunization coverage in Ethiopia was 52.3% (95% CI: 50.3, 54.3). The immunization coverage varied substantially at the sub-national level with spatial clustering of low immunization coverage observed in the Eastern, Southeastern, and Northeastern parts of Ethiopia. The spatial clustering of the rotavirus immunization coverage was positively associated with altitude of the area [mean regression coefficient (β): 0.38; 95% credible interval (95% CrI): 0.18, 0.58] and negatively associated with travel time to the nearest cities in minutes [mean regression coefficient (β): − 0.45; 95% credible interval (95% CrI): (− 0.73, − 0.18)] and distance to the nearest health facilities [mean regression coefficient (β): − 0.71908; 95% credible interval (95% CrI): (− 1.07, − 0.37)].

**Conclusions:**

This study found that the rotavirus immunization coverage varied substantially at sub-national and local levels in Ethiopia. The spatial clustering of rotavirus immunization coverage was associated with geographic and healthcare access factors such as altitude, distance to health facilities, and travel time to the nearest cities. The immunization program should be strengthened in Ethiopia, especially in the Eastern, Southeastern, and Northeastern parts of the Country. Outreach immunization services should be also implemented in areas with low coverage.

**Supplementary Information:**

The online version contains supplementary material available at 10.1186/s12879-022-07825-1.

## Introduction

Diarrheal diseases are the second leading cause of death among under-five children globally [1–6], claiming more than 484,000 death and 2.6 million illnesses in 2016 [7]. Rotavirus is the most common cause of severe diarrheal disease, which is associated with 128, 500 deaths [8, 9]. Ethiopia is among the five countries with the highest rotavirus burden accounting for six percent of the global rotavirus deaths [10]. The World Health Organization (WHO) introduced an effective two-dose vaccine to prevent diarrheal disease caused by rotavirus [7, 11, 12]. Ethiopia adopted this recommendation and introduced the Rota vaccine into the national immunization program in 2013 [10]. However, the coverage remains low in Ethiopia and below the 75% national immunization coverage target [13, 14].

According to the WHO report, the estimated rotavirus immunization coverage was 46% globally, and 50% in Africa in 2020 [15, 16]. The Ethiopian Demographic and Health Survey (EDHS) reported that the national rotavirus immunization coverage was nearly 47% in 2016 [17–19]. Further regional level analysis of the 2016 EDHS showed that rotavirus immunization coverage varied from 14.6% in Afar to 77% in Addis Ababa [18]. However, the rotavirus immunization coverage for lower administrative units such as districts may differ significantly from the regional and national average [14]. Measuring rotavirus immunization coverage in a lower geographic area can provide information for designing and implementing a targeted immunization campaign [20, 21].

Understanding drivers of the spatial distributions of rotavirus immunization coverage are also essential to inform strategies for improving immunization services. Previous studies have identified various factors that affect different immunization coverage such as measles and influenzas. These factors include climatic conditions and access to health facilities [22–27]. However, the impacts of these factors on rotavirus immunization coverage are yet to be quantified in Ethiopia [28]. Therefore, this study aimed to estimate the spatial distributions and identify drivers of rotavirus immunization coverage in Ethiopia.

## Methods

### Study setting

The study was conducted in Ethiopia which is located in east Africa. Ethiopia has a surface area of approximately 1.1 million square kilometers. The country has a variety of geographical features with altitudes ranging from 125 m below sea level in the Danakil depression, Afar region to 4620 m above sea level in Ras Dajen mountain, Amhara region. Ethiopia is the second-most populous country in Africa with an estimated population size of more than 115 million people in 2020. Of these populations, 16.8 million are children under five years of age [29].

### Immunization program in Ethiopia

The rotavirus immunization was one of the lifesaving vaccines given to children as part of the national immunization program in Ethiopia since 2013 [10]. More than half of the population in Ethiopia lives more than 10 km far from the nearest health facilities, which might affect the immunization programs [30]. The Ethiopia health care system has a three-tier system: (1) primary care: composed of health posts, health centers, and primary hospitals; (2) secondary care: composed of general hospitals; and (3) tertiary care: composed of specialized hospitals [31]. Immunization services are delivered at all levels.

### Data sources

Rotavirus immunization coverage data with geographic coordinates were obtained from the mini-Ethiopian demographic and health survey (MEDHS-2019). The EDHS-19 survey was conducted between January and June 2019. The survey contains data that is relevant to estimating rotavirus immunization coverage at a pixel level. The outcome variable for this study is full rotavirus immunization coverage, which is defined as the number of children vaccinated with two doses of rotavirus divided by the total number of children included in the survey. A polygon shapefile for the Ethiopian administrative boundaries was obtained from the central statistical agency of Ethiopia-2013. Climatic variables such as mean annual temperature and mean annual precipitation were obtained from the WorldClim website [32]. Altitude data were obtained from the Shuttle Radar Topography Mission (SRTM) [33]. Data on travel time to the nearest city and travel times to the nearest healthcare facility in minutes (i.e., hospital or clinic) were obtained from the Malaria Atlas Project (MAP) [34]. Population density, estimated as the number of people per grid cell, was obtained from WorldPop [35]. Rotavirus immunizations coverages data were linked to area-level covariates data by using ArcGIS software.

### Spatial analysis

The spatial continuous estimates of the national rotavirus immunization coverage map were generated by using Bayesian model-based geostatistics at a resolution of 1 km^2^. A spatial binomial regression model was fitted for rotavirus immunization coverage survey data including fixed effects for altitude, travel time to the nearest city, distance to the nearest health facilities and population density, and geostatistical random effects [36]. The rotavirus immunization coverage was taken at each surveyed location *j* as the outcome variable, which was assumed to follow a binomial distribution:

$${Y}_{j}\sim Binomial \left({n}_{j},{p}_{j}\right);$$

where ${Y}_{j}$ is the proportion of children vaccinated for rotavirus, ${n}_{j}$ is the number of children vaccinated for rotavirus and ${p}_{j}$ is the predicted rotavirus immunization coverage at location $j$(j = 1, …305). Mean predicted rotavirus coverage was modeled via a logit link function to a linear predictor defined as:

$$logit \left({p}_{j}\right)=\alpha +{\sum }_{z=1}^{z}{\beta }_{z}{{\varvec{X}}}_{z,j }{+ }{\zeta }_{j};$$

where *α* is the intercept, *β* is a matrix of covariate coefficients, ${\varvec{X}}$ is a design matrix of $z$ covariates, and ${\zeta }_{j}$ are spatial random effects modeled using a zero-mean Gaussian Markov random field (GMRF) with a Matérn covariance function. The covariance function was defined by two parameters: the range $\rho$, which represents the distance beyond which correlation becomes negligible (about 0.1), and $\sigma$, which is the marginal standard deviation [37, 38]. Non-informative priors were used for *α* (uniform prior with bounds − ∞ and ∞) and we set normal priors with mean = 0 and precision (the inverse of the variance) = 1 × 10^–4^ for each *β*. We used default priors for the parameters of the spatial random field [39]. Parameter estimation was done using the Integrated Nested Laplace Approximation (INLA) approach in R (R-INLA) [37, 38]. Sufficient values (i.e., 150,000 samples) from each simulation run for the variables of interest were stored to ensure full characterization of the posterior distributions.

Predictions of rotavirus immunization coverage at unsampled locations were made at 1 km^2^ resolution by interpolating the spatial random effects and adding them to the sum of the products of the coefficients for the spatially variant fixed effects at each prediction location [40]. The intercept was added, and the overall sum was back-transformed from the logit scale to the prevalence scale, providing prediction surfaces that show the estimated rotavirus immunization coverage for all prediction locations. The Watanabe Applicable Information Criterion (WAIC) statistic was used to select the best-fitting model.

## Results

### National and regional rotavirus immunizations coverage

Table 1 shows the national and regional rotavirus immunization coverage in Ethiopia. The national rotavirus full immunization coverage was 52.3% (95% CI: 50.3, 54.3). Substantial variation was observed at regional levels, with the highest immunization coverage in Addis Ababa (91.67%), Tigray (70.77%), and Dire Dawa (70.39%). On the other hand, low immunization coverage was observed in Afar (24.1%) and Somali (26.42%) regions.

**Table 1:** National and regional rotavirus immunization coverage in Ethiopia in 2019

Region	Rota 1 immunizations coverage %	Rota 2 immunizations coverage %
Afar	31.03	24.1
Somali	33.02	26.42
SNNPR	52.74	42.65
Gambela	59.67	51.03
Harari	60.8	46
Oromia	62.5	52.81
Benishangul-Gumuz	70	61.79
Amhara	75.78	68.86
Tigray	78.08	70.77
Dire Dawa	81.12	70.39
Addis Ababa	92.78	91.67
Ethiopia	60.77	52.33

*SNNPR* Southern Nations, Nationalities and peoples Region

### Spatial clusters of rotavirus immunizations

**Fig. 1:**
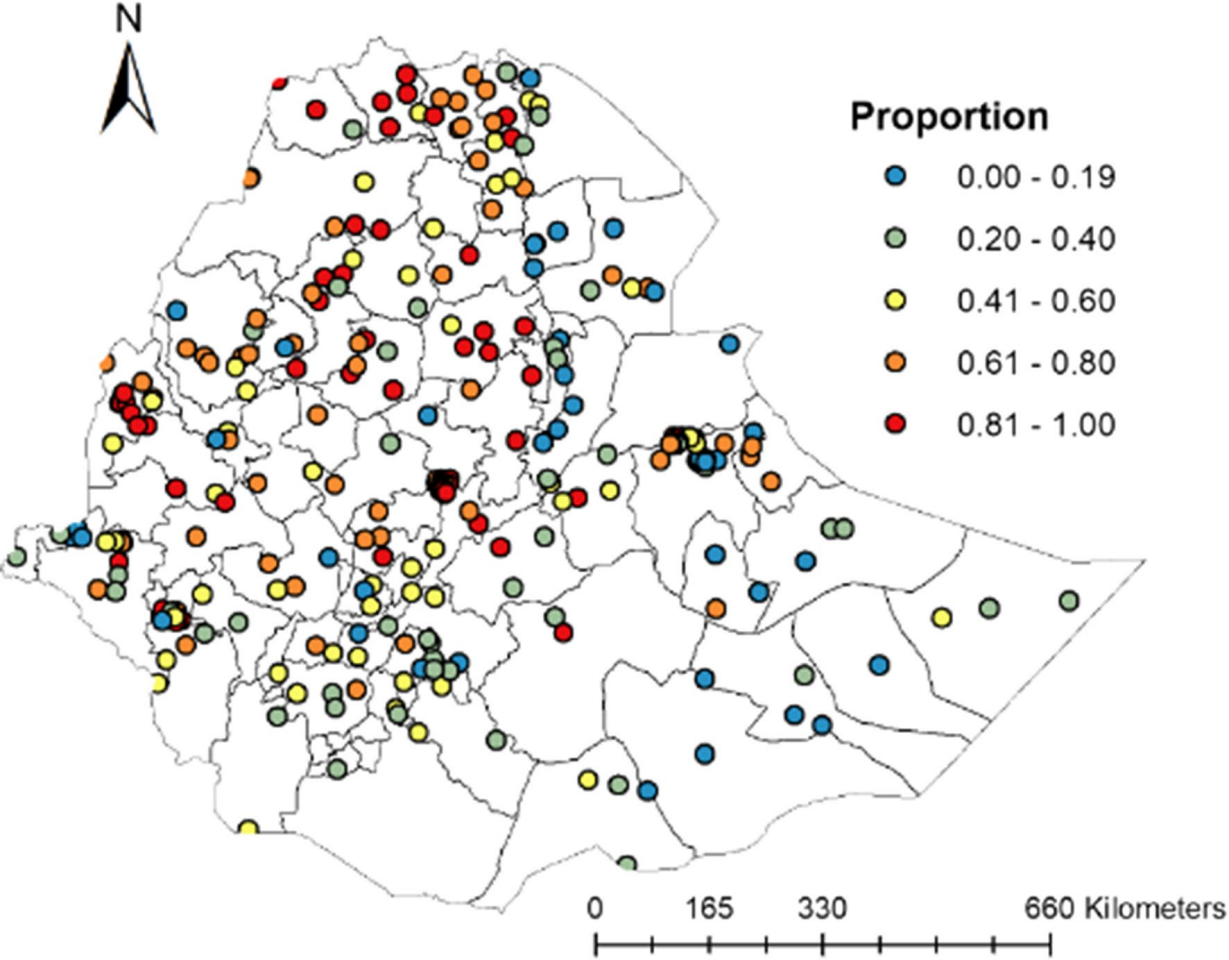
Geographical locations of data points and rotavirus immunization coverage in Ethiopia

The rotavirus immunization coverage substantially varies within regions of Ethiopia. Spatial clustering (i.e., highest rotavirus immunization coverage) of high coverage was observed in the Central, Northern, and Northwestern parts of Ethiopia. On the contrary, spatial clustering of low rotavirus immunization coverage was seen in the Southern, Southeastern, and Eastern Northeastern parts of the country (Fig. 1).

**Fig. 2:**
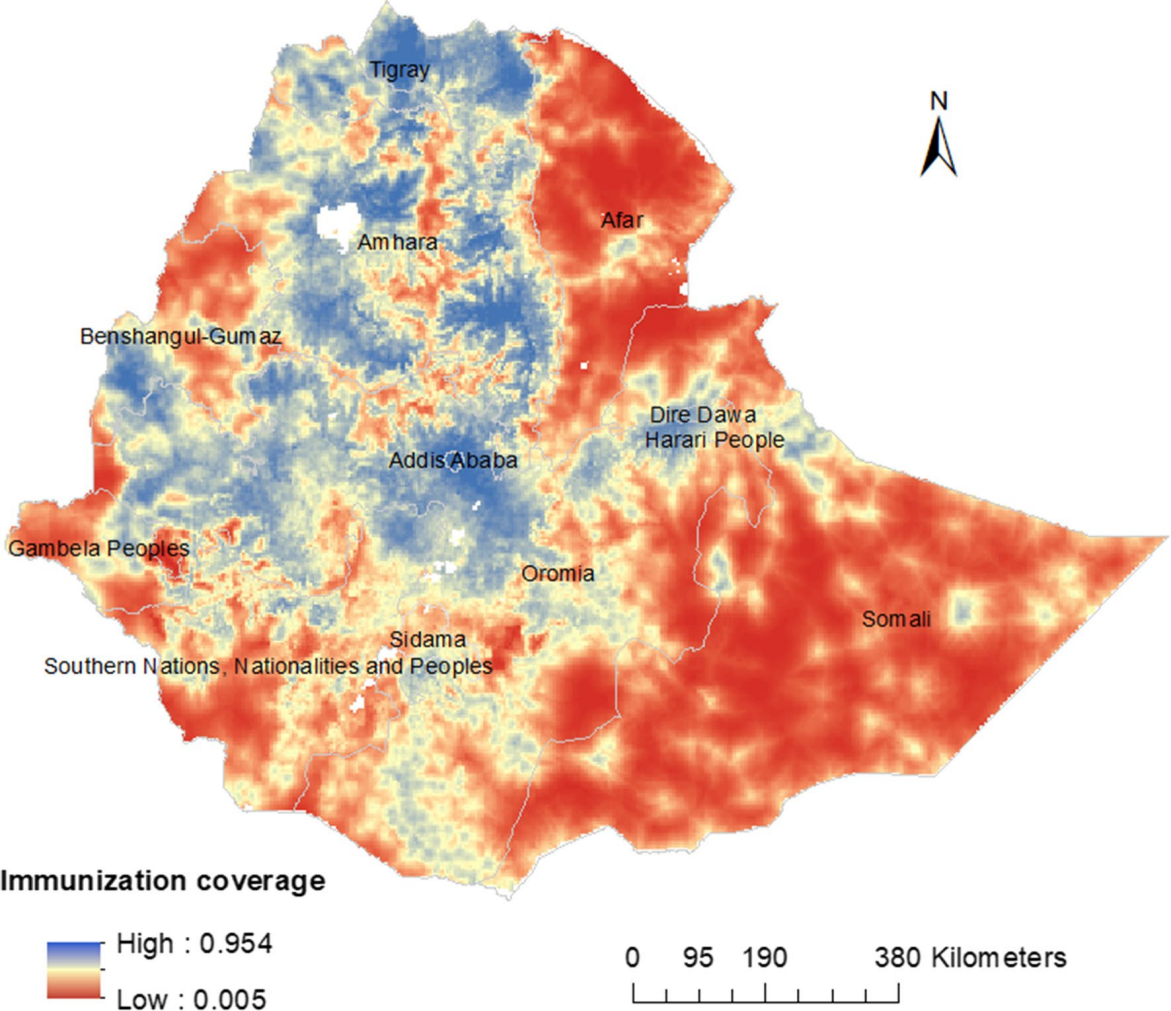
The predicted geospatial map for rotavirus immunization coverage in Ethiopia

Figure 2 showed the predicted map of immunization coverage in Ethiopia using the Bayesian model framework. Predicted low rotavirus immunization coverage was observed in Southern, Eastern, and Northwestern parts of the country. Whereas, the highest predicted immunization coverage was observed in the Northern, Central, and Northeastern parts of Ethiopia.

### Drivers of rotavirus immunizations coverage in Ethiopia

The Bayesian geostatistical model found that geographic and climatic factors were associated with the spatial clustering of rotavirus immunization coverage in Ethiopia. The altitude of the area in meters [mean regression coefficient (*β*): 0.38; 95% credible interval (95% CrI): 0.18, 0.58] was positively associated with rotavirus immunization coverage. Whereas, travel time to the nearest cities in minutes [mean regression coefficient (*β*): − 0.45; 95% credible interval (95% CrI): (− 0.73, − 0.18)] and walking distance to health facilities in minutes [mean regression coefficient (*β*): − 0.719; 95% credible interval (95% CrI): (− 1.07, − 0.37)] were negatively associated with rotavirus immunization coverage in Ethiopia (Table 2).

**Table 2:** Regression coefficient mean and 95% credible intervals (CrI) of covariates included in a Bayesian spatial model with Binomial response for the rotavirus immunization coverage in Ethiopia, 2019

Covariates	Rotavirus immunization coverage Regression coefficients of Mean (95% CrI)
Altitude	**0.38 (0.18, 0.58)**
Travel time	**− 0.45 (− 0.73, − 0.18)**
Population density	0.003 (− 0.008, 0.01)
Distance to health facilities	**− 0.718 (− 1.07, − 0.37)**
Intercept	− 0.96 (− 1.32, − 0.62)

CrI: credible interval; bold fonts show ‘statistically significant’ results within a Bayesian framework (no zero within the 95% CrI)

Model fitness was checked by using the Widely Applicable Information Criterion (WAIC) statistic, the model that contained the smallest value (i.e., the model which contain all covariates) was the best-fitting model for immunization coverage (Additional file 1: Table S1).

## Discussion

Rotavirus immunization coverage was 52.3% (95% CI: 50.3, 54.3) in Ethiopia, which is slightly higher than the rotavirus immunization coverage reported in the 2016 EDHS. The rotavirus immunization coverage was also higher than the global (46%) [41] and Africa (50%) [16] estimates. This might be because of the strong commitment of the Ethiopian government in the past few years to improve access to and utilization of immunizations through training a large number of health extension workers, which deliver vaccines at the community level both in rural and urban districts. Moreover, expansion of health posts and primary health cares in the country may provide vaccines and increase coverage. However, rotavirus immunization coverage in our study is lower than a study conducted in developed countries such as the USA (60.4%) [27] and Canada 84% [42]. This might be due to the difference in socioeconomic development, accessibility and utilization of health services since most of the population in sub-Saharan Africa including Ethiopia live in rural areas which are underserviced community settings [43]. It could also be due to the difference in community awareness towards vaccine programs and differences in infrastructure including distance to health facilities [44, 45]. The accessibility and utilization of health services including immunization services in Ethiopia are unevenly distributed. The health service is low in the rural parts of Ethiopia, where greater than 80% of the population live.

Consistent with other spatial studies [46, 47], the current study revealed significant spatial clustering of rotavirus immunization in Ethiopia. Spatial clustering of low rotavirus immunization coverage was observed in the Eastern, Southeastern, and Northeastern parts of the county. This might be due to the low socioeconomic status of the household, low healthcare-seeking behaviors, inadequate knowledge of childhood immunization, poor access to vaccines, and misconceptions about immunization in pastoral regions like Somalia and Afar regions [48, 49].

Our study found that altitude was positively associated with rotavirus immunization coverage. This might be because most kebeles in the highland area have access to transportation. Moreover, districts in the highland areas have relatively better healthcare access than districts in lowland areas such as in Afar and Somali regions [45, 50]. In addition, due to the absence of infrastructure in these areas, missed opportunities due to vaccine stock out could contribute to the low coverage of rotavirus immunization [51].

Travel time to the nearest cities and distance to health facilities were negatively associated with rota vaccine coverage. This finding is consistent with previous pieces of studies conducted in Ethiopia [45, 50, 52] and other African countries such as Tanzania [53], Malawi [54], Nigeria [55], and Kenya [56]. This might be because access to health facilities and poor infrastructure are the major challenges for healthcare providers to provide proper healthcare services including immunization [57]. In addition, if there is no access to health facilities, mothers and caregivers of a child might not visit health facilities for immunization services. Moreover, people living away from the cities and health facilities have low access to information and health education which could, in turn, lead to poor health care utilization including child immunization [58]. Designing and implementing a targeted immunization campaign in areas with low rotavirus immunization coverage would be important.

This study provides important information that would help to minimize the geographic disparity of rotavirus immunization uptake in Ethiopia. The study identified high-risk areas and underline factors of rotavirus immunization coverage in Ethiopia. Such information is important for integrated intervention to achieve Sustainable Development Goal targets for child survival. Immunization has a significant impact on advancing sustainable development goals [59]. Its contribution to building a productive workforce (SDG8) makes it to be the main driver of the economic development of a given country [60].

Furthermore, as a strategy, the reaching every child approach was first introduced in Ethiopia in 2003 and populations at risk of missing immunization services are the primary focus of the immunization policy of Ethiopia [61]. Therefore, knowledge of the specific geographic areas of low rotavirus vaccine uptake has practical implications and is valuable input to the health system of Ethiopia. While there is significant maternal and child health services progress at the national level in Ethiopia, the geographic disparity and inequity of these services are still policy priority areas [62]. Therefore, the findings in this study could serve as a potential source of evidence for policymakers and program designers.

The strength of this study was using nationwide data which could produce reliable estimates with advanced geostatistical analysis. However, this study had some limitations, due to the cross-sectional nature of the data, it might be difficult to indicate the temporal relationship between geospatial covariates and the outcome variable. In addition, important demographic factors such as residence status, community literacy, and institutional delivery services were not included due to a lack of data.

## Conclusion

This study found that the national immunization coverage varied substantially at sub-national and local levels, suggesting that interventions could be geographically targeted. Spatial clustering of low rotavirus immunization coverage was observed in the Eastern, Southeastern, and Northeastern parts of Ethiopia. Access to health facilities and geographic factors including distance to health facilities and travel time to cities were associated with the low rotavirus immunization coverage in Ethiopia. This finding suggests that intervention programs targeting populations with significant distance to health facilities and cities be more cost-effective than a generic approach. The health extension program in Ethiopia should implement outreach immunization campaigns in areas with low immunization coverage. Community advocacy and mobilizations should be also strengthened especially in low land areas to improve immunization coverage in Ethiopia.

## Supplementary Information

**Additional file 1. Table S1**. Watanabe-Akaike information criterion (WAIC) values corresponding to different model specifications.** Figure S1**. standard deviation of the immunization coverage.

## Data Availability

Relevant data is included within the manuscript and Additional file 1. If additional data is needed, it could be accessed by contacting the corresponding author.

## Abbreviations

EDHS: Ethiopian Demographic and Health survey
GMRF: Gaussian Markov random field
INLA: Integrated Nested Laplace Approximation
MAP: Malaria Atlas Project
MEDHS: Mini Ethiopian Demographic and Health Survey
SRTM: Satellite Radar Topography Mission
WAIC: Watanabe applicable information criterion
WHO: World Health Organization
