# Supplementary material for: Spatial distribution of rotavirus immunization coverage in Ethiopia: a geospatial analysis using the Bayesian approach
Source: BMC Infect Dis. 2022 Nov 9;22:830. doi: 10.1186/s12879-022-07825-1 (PMC9648028; doi:10.1186/s12879-022-07825-1)
Supplement: Supplementary file 1 — Additional file 1. Table S1. Watanabe-Akaike information criterion (WAIC) values corresponding to different model specifications. Figure S1. standard deviation of the immunization coverage. [file 12879_2022_7825_MOESM1_ESM.docx]

**Supplementary files**

**Table S1:** Watanabe-Akaike information criterion (WAIC) values corresponding to different model specifications.

| Model specifications | WAIC |
| --- | --- |
| Altitude | 1212.112 |
| Altitude + Travel time | 1192.058 |
| Altitude + Travel time + Pop | 1198.543 |
| Altitude + Travel time + Population density + Distance from Health Facilities | 1182.122 |


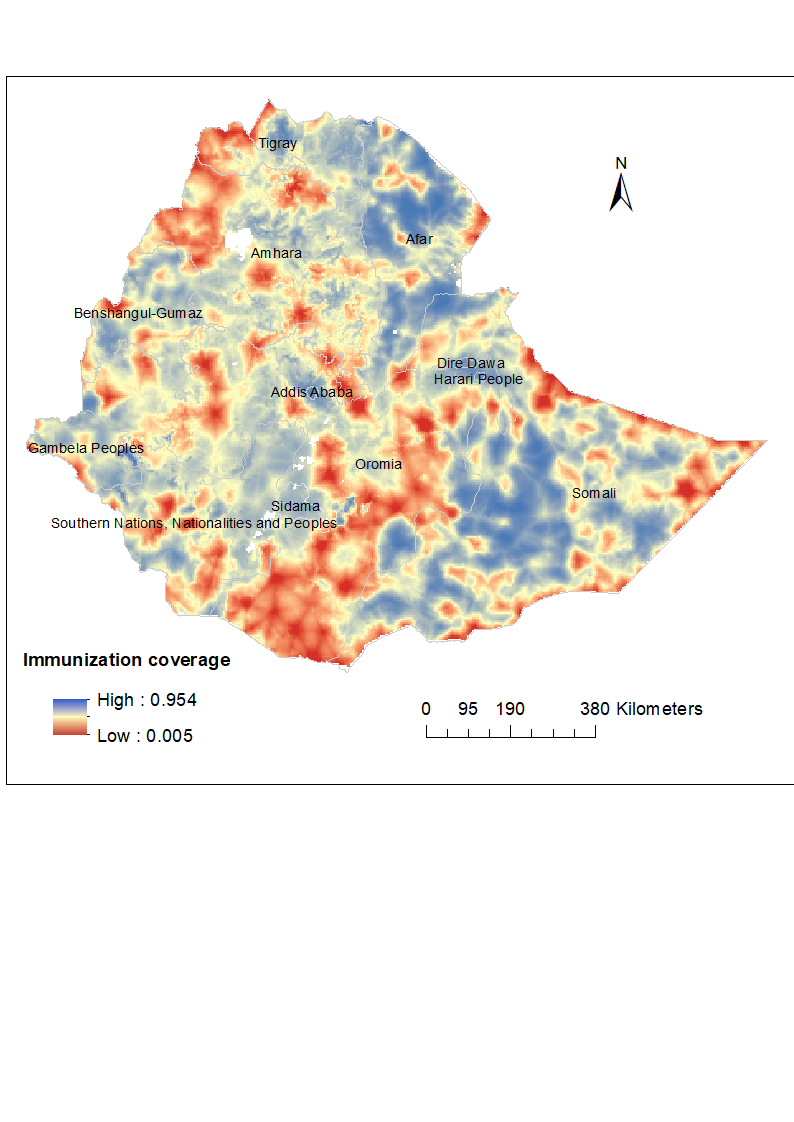


**Figure S1:** standard deviation of the immunization coverage
